# Editorial—Cholera Observations

**Published:** 1893-08

**Authors:** 


### EDITOR'S OUTLOOK.

### CHOLERA OBSERVATIONS.

Realizing that cholera is prevailing in the "old world," and any day is liable to be brought to our own shores, despite the fact that every precaution is being taken to prevent it, we feel it our duty towards humanity to say a word. Suppose our medical profession, at this time, with the aid of the press, arise and make a combined movement to help the community to an accurate discrimination of the disease in its early stages. The distinction between Asiatic cholera and common diarrhœa is palpable and easy, and every man can carry that distinction in his memory. Cannot an uneducated man tell certainly if he has an evacuation which is *copious*, *watery*, *colorless*, *painless* and *inodorous?* Any man of ordinary talents can ascertain, in two minutes, that something has happened to him which he never experienced before. I said *painless.* It is this quality of the evacuation which leads men to the amazing apathy so common, and permits them to let hours—even days—elapse before the physician is at his post. As this Asiatic destroyer has now become Americanized, our people *must* be able to make an early discrimination, and our profession *must* learn how to prevent the fatal collapse. Our formula in the early stage of cholera is a simple one, and should be borne in mind by every reader of the JOURNAL. Combine 15 grains of calomel and 4 grains of opium. Take every four hours. If every business man would keep a powder of the above prescription in his pocket, to swallow if occasion required, it would scarcely do harm and would greatly aid the efforts of the physician employed.

---

In our September issue, our Summer Recreation Bureau will be discontinued until another year.

We realize that we have done a great work, and been the means of aiding many people by practical suggestions. Our Educational Bureau will be inaugurated in the October number, and any of our readers who desire a catalogue of any school of prominence in the United States or Canada, make your wishes known by addressing our Educational Bureau.

---

Another month remains for those who desire to take advantage of our Encyclopædia offer. Last chance.

